# Protective effects of honey by bees (*Apis dorsata*) on decreased cortical thickness and bone impact strength of ovariohysterectomized rats as models for menopause

**DOI:** 10.14202/vetworld.2019.868-876

**Published:** 2019-06-22

**Authors:** Ira Sari Yudaniayanti, Hardany Primarizky, Lianny Nangoi, Gandul Atik Yuliani

**Affiliations:** 1 Department of Veterinary Clinic, Faculty of Veterinary Medicine, Universitas Airlangga, Kampus C Unair, Mulyorejo, Surabaya 60115, Indonesia; 2 Department of Basic Veterinary Medicine, Faculty of Veterinary Medicine, Universitas Airlangga, Kampus C Unair, Mulyorejo, Surabaya 60115, Indonesia

**Keywords:** bone impact strength, cortical thickness, honey *Apis dorsata*, microarchitecture, ovariohysterectomy

## Abstract

**Aim:**

This study aimed to determine the potential of honey as anti-osteoporosis by evaluating its effectiveness in increasing bone impact strength and cortical thickness, through scanning electron microscopy (SEM) examination.

**Materials and Methods:**

Forty-five female rats at 3 months of age, weighing 150-200 g were used in the study. They were placed in individual cages and adapted to food and environment for 10 days. On the 11^th^ day, after the animals were adapted for 10 days, the animals were randomly divided into five treatment groups (n=9): Sham operation group (SH); ovariohysterectomized (OVX) group with no treatment; OVX with treatment *Apis dorsata* 1 g/kg BW (AD-1); OVX with treatment *A. dorsata* 2 g/kg BW (AD-2); and OVX with treatment *A. dorsata* 4 g/kg BW (AD-3). Furthermore, those nine rats in each treatment group were divided into three groups. Three of them were observed at months 1^st^, 2^nd^, and 3^rd^ so that in each observation taken three rats in each treatment group. At the end of the study, the rats were euthanized and necropsy for taking their second femoral bone, i.e. dexter region for examining their bone impact strength, while the sinister region was used for measure the cortical thickness of the femoral diaphysis and examining their bone microarchitecture using SEM analysis.

**Results:**

Based on results of the ANOVA test, the cortical thickness measurements of femoral diaphyseal can be seen that from month 1 to month 3 the lowest result was found in the group of rats that were OVX-I. Meanwhile, the highest result was found in the group of rats that were not OVX (SH-III). It was significantly different from the other treatment groups (p<0.05). The groups of rats were OVX with honey supplementation at doses of 2 g/kg BW had shown an increasing pattern in the cortical bone thickness from month 1 to month 3. Even on the observation of the 3^rd^ month, the cortical bone thickness in the AD-2 (AD-2-III) group was not significantly different (p>0.05) from that in the group of rats was not OVX in month 1 (SH-I). The results of the bone impact strength measurement from month 1 to month 3 indicated that the groups of rats were OVX without the administration of honey supplements had the lowest value. The highest bone impact strength was found in the group of rats that was not OVX, but not significantly different (p>0.05) with the groups of rats that were OVX administered honey supplement with a dose of 2 g/kg BW (AD-2) and 4 g/kg BW (AD-3).

**Conclusion:**

The supplement of honey *A. dorsata* at doses of 2 g/kg BW in the group of rats was that OVX can inhibit the decreasing of the cortical bone thickness and repair damage in microarchitecture to generate bone impact strength. As a result, bones are not easily broken.

## Introduction

Osteoporosis has currently been considered as a global health problem. Osteoporosis even has been included in the top 10 degenerative diseases in the world by the World Health Organization. There are approximately 200 million women worldwide suffer from osteoporosis [1]. Based on data analysis by the Department of Health in Indonesia together with Fonterra Brands Indonesia, the prevalence of osteoporosis in Indonesia reached 41.75% in 2006, that is, every two from five of Indonesia’s population are at risk for osteoporosis [2]. Osteoporosis is always associated with an increased risk of fracture 2-3 fold compared to normal bone in the event of mild trauma. Besides, osteoporosis has contributed to fracture, disability, dependence on others, and psychological disturbances, thereby decreasing the quality and function of life as well. Osteoporosis is also referred to as a silent disease since bone density process reduces gradually and progressively for years without being realized due to having no symptoms. Patients with osteoporosis that can be detected before a fracture are only <25% [3].

The occurrence of osteoporosis is basically due to the excessive amount and activity of osteoclasts higher than the amount and activity of osteoblasts (bone-forming cells). There are several theories revealing factors causing osteoclast differentiation and activity to increase, one of which argued it is partly due to estrogen deficiency. Geng *et al*. [4] suggested that estrogen deficiency would lead to an increase in oxidative stress that can reduce osteoblastic bone formation and also related with increased osteocyte senescence and secretory phenotype, while it can enhance osteoclastic differentiation and ultimately lead to more bone resorption. The decrease in estrogen levels is usually common in menopausal women and also in animals which have ovariohysterectomy (OVX) or ovariectomy with the aim of sterilization and medical action for handling cases of reproductive diseases [5]. Although estrogen deficiency is a major cause of osteoporosis, the use of estrogen supplementation/hormone replacement therapy (HRT) for both treatment and prevention purposes is not recommended because long-term HRT use increases levels of sex hormones in the circulation, thus increasing the risk of breast cancer, endometrial cancer, thromboembolic events, and vaginal bleeding [6]. Current osteoporosis therapies mainly concerned with anti-resorption, which may be seriously related side effects, such as the attenuation of bone formation. Therefore, it is necessary to quest alternative therapies (natural) that have minimal side effects. Currently, medicines with natural ingredients are efficacious as an alternative choice because herbal medicines are cheaper and safer than synthetic chemical drugs [7]. Research on the utilization of natural ingredients for the prevention and treatment of osteoporosis has also been widely conducted [8]. One of the natural ingredients used in increasing bone density is honey [9]. Honey has also been known to contain anti-osteoporosis [9-11]. Honey with α-Cyclodextrin, according to a research report conducted by Katsumata *et al*. [12], has a prebiotic effect that can decrease bone tissue resorption. Honey contains flavonoid compounds, more than 150 polyphenol compounds, including phenolic acids, flavonoids, flavonols, catechins, and cinnamic acid derivatives [13]. The mechanism of action of flavonoids in protecting bone tissue is by reducing bone loss through antioxidants. In addition, flavonoids relieve the inflammatory response as an anti-inflammatory. Flavonoids also work on a bone turnover by increasing osteoblastogenesis, suppressing osteoclastogenesis, as well as through osteoimmunological action [14]. Honey combined with a jumping exercise can increase alkaline phosphatase and calcium serums as bone metabolism markers; therefore, its consumption is recommended for sportsmanship [11].

There actually have been many researches on honey, but the number of researches on honey related bone metabolism and characterization in cases of osteoporosis is still lacking. In fact, honey forest production, *Apis dorsata*, in Indonesia is high as much as 70% [15]. As a result, this research aimed to analyze the potential of honey in the study of medical biology as anti-osteoporosis by evaluating its effectiveness in increasing bone impact strength and cortical thickness, through scanning electron microscopy (SEM) examination. Thus, the results of this research are expected to be used to prevent bone fractures due to osteoporosis. This study used rats as experimental animals. Rats have been widely accepted as animal models for studying bone diseases such as the process of remodeling and bone resorption because the responses of bone toward mechanical stress, hormones, and drugs are similar in rat and human [16]. Therefore, the ovariectomized rat is a useful model for postmenopausal osteoporosis [17].

This study aimed to analyze the potential of honey bees (*Apis dorsata*) as anti-osteoporosis by evaluating its effectiveness in increasing bone impact strength and cortical thickness.

## Materials and Methods

### Ethical approval

This study was approved by the Animal Ethics Committee of Faculty of Veterinary Medicine Universitas Airlangga (No. 665-KE/March/2017).

### Study design

This research used female Wistar strain rats (*Rattus norvegicus*) as experimental animals aged 3 months old and weighed 150-200 g. The number of rats used was 45 rats. Those rats were obtained in healthy condition from the Faculty of Medicine, Universitas Airlangga. Those experimental animals then were given certain treatments. They were placed in individual cages and adapted to food and environment for 10 days. During the adaptation period, those rats were fed with standard food as much as 10% of their body mass (± 20 g) and provided with *ad libitum* drinking of water. On the 11^th^ day, after the animals were adapted for 10 days, the animals were randomly divided into five treatment groups, each of which consisted of nine rats. Those nine rats in each treatment group then were divided into three groups with different examination stages. Those five treatment groups were given different treatment as follows:

- Sham-operated (SH) group, not ovariohysterectomized, but given aquades using probe from day 1 after the surgery
- OVX group, ovariohysterectomized and then given aquades using probe from day 1 after the OVX
- AD-1 group, ovariohysterectomized, and then given aquades as well as bee honey (*A. dorsata*) using probes as much as 1 g/kg BW from day 1 after the OVX
- AD-2 group, ovariohysterectomized, and then given aqudes as well as bee honey (*A*. *dorsata*) using probes as much as 2 g/kg BW from day 1 after the OVX
- AD-3 group, ovariohysterectomized, and then given aqudes as well as bee honey (*A*. *dorsata*) using probes as much as 4 g/kg BW from day 1 after the OVX.

Furthermore, those nine rats in each treatment group were divided into three groups. Three of them were observed at months 1^st^, 2^nd^, and 3^rd^ so that in each observation taken three rats in each treatment group.

### Making of osteoporosis model

Making osteoporosis model was performed in rats in the OVX, AD-1, AD-2, and AD-3 groups through bilateral OVX. It was analogical with dogs or cats that have been OVX, as well as women who have menopause, where estrogen deficiency occurs in both conditions. Before surgery, the weight of each rat was measured to determine the dose of anesthesia. Subsequently, those rats were anesthetized using a combination of ketamine (50 mg/kg BW) and xylazine (10 mg/kg BW) [18]. This procedure of OVX was performed in accordance with a method proposed by Delaney [19]. Furthermore, 1 day after the OVX, all rats were treated in accordance with the group that had been determined. This research lasted for 3 months, divided into three observations. During the adaptation, until the end of the treatments, all groups of animals were given standard feed. At the end of the study, the rats were euthanized and necropsy for taking their both femoral bone, i.e. Dexter region for examining their bone impact strength, while the sinister region was used for measure the cortical thickness of the femoral diaphysis and examining their bone microarchitecture using SEM analysis.

### SEM analysis

SEM was an analytical method used to characterize sample surfaces. Its working principle was based on the electron beam focused with the electromagnetic lens to the sample surface. Before SEM characterization, sample preparation then was performed using grind levels of 120, 400, 800, and 1200 gradually. After grinding the samples, MicroPolish alumina sampling with a size of 0.3 µ, 1 µ, and 5 µ gradually was conducted using a grinding machine. Samples then were observed with an optical microscope to see their surface state until they could be continued for preparation. Next, H_2_O was removed from the samples. After the samples were dry, the samples were affixed to specimen holder and then cleaned by hand blower. Afterward, the samples were coated with gold-palladium because the samples were not a metal type that can emit electrons. Once the samples were ready, they then were put into the specimen chamber to be observed and analyzed for their micro structure on the SEM screen. Results of the SEM image then would indicate their topography (their surface texture) and morphology (shape and size of particles arranging them).

### Measurement of bone impact strength

First, the test specimens were measured in dimensions using calipers or sliding range. All results obtained then were recorded in a table. Second, the test piece was placed on the anvil in a horizontal position, while notch was placed right in the center of the specimen. Third, the notch was faced in a parallel direction to the arrival of shock load. Fourth, the shock load was raised at a large angle (where the angle α is taken depends on the specimen’s resistance ± 45°-90°). Fifth, the initial position of the needle was adjusted, and the initial scale pointer was at zero. Sixth, the shock load was removed. After hitting the test piece, the shock load would still swing with a large β angle that should be observed as well as noted in the observation table.

Meanwhile, energy used to break the test piece was seen on the needle of the pointer scale and recorded in the observation table. This energy can theoretically also be calculated based on a formula. The impact strength of the test piece was calculated by dividing the total energy to break the test piece by the cross-sectional area of the affected part. Moreover, finally, bone impact strength was calculated by the following formula:


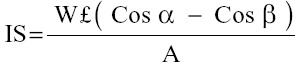


Note:

β = final angle

α = initial angle

A = test sectional area of test specimen (mm^2^)

W = weight of shock load (gr)

£ = length of shock load arm (mm)

### Statistical analysis

After the data were obtained, they were tabulated and then tested with both Kolmogorov–Smirnov test (p>0.05) for analyzing their normality and Levene test (p>0.05) for evaluating their homogeneity. Next, the other quantitative data obtained in this research, bone impact strength and cortical thickness of those rats, were analyzed using SPSS 17.0 for Windows software (SPSS, Chicago, IL, USA). Differences between treatment groups then were evaluated using ANOVA, followed by Duncan multiple range test.

## Results

### Phytochemical screening test on forest honey (*A. dorsata*)

Based on the results of the phytochemical screening tests, forest honey (*A. dorsata*) contains several active ingredients, namely alkaloids, triterpenoids/free steroids, unsaturated steroids, flavonoids, polyphenols, and tannins (Table-1). It indicates that forest honey (*A. dorsata*) contains an almost all active ingredients, very useful for bone formation and growth.

**Table-1:** Phytochemical components of forest honey (*Apis dorsata*).

Phytochemical components	Results
Triterpenoid/free steroid	+
Unsaturated steroid	+
Flavonoid	+
Polyphenol	+
Tannin	+

### SEM examination on the microarchitecture and femoral cortical bone thickness of ovariohysterectomized rats (*Rattus norvegicus*)

The SEM examination was conducted to observe the microarchitecture of the femoral cortical bone of the ovariohysterectomized rats. This SEM examination involved the measurement of femoral cortical bone thickness and the observation of damaged area considered as important parameters in determining bone quality.

The results of the cortical thickness measurements of femoral diaphyseal are shown in Table-2. Based on the results of the ANOVA test, it can be seen that from month 1 to month 3 the lowest result was found in the group of rats that were OVX-I. It was significantly different from the other treatment groups (p<0.05). Meanwhile, the highest result was found in the group of rats that were not OVX (SH-III). It was significantly different from the other treatment groups (p<0.05). The results of this research also showed that there was a significant difference in the decreasing of the cortical thickness of the femoral diaphysis in the group of rats that were OVX every month (p<0.05).

**Table-2:** Mean and standard deviation of the cortical thickness measurements (mm) observations of 1^st^, 2^nd^, and 3^rd^ months in all treatment rat (*Rattus* *norvegicus*) groups.

Group	1^st^ month	2^nd^ month	3^rd^ month
SH	510.48^hi^±12.01	531.21^i^±14.46	532.29^i^±11.10
OVX	331.85^a^±14.83	286.55^b^±13.19	219.56^c^±14.48
AD-1	391.75^d^±14.61	376.57^d^±11.88	376.93^d^±11.88
AD-2	449.79^ef^±13.36	470.23^fg^±22.65	488.89^gh^±12.99
AD-3	469.2^fg^±11.43	467.57^fg^±12.89	438.17^e^±14.05

a,b,c,d,e,f,g,h,i different superscripts in the same column and row indicate significant differences (p<0.05). OVX: Ovariohysterectomy

**Figure-1:**
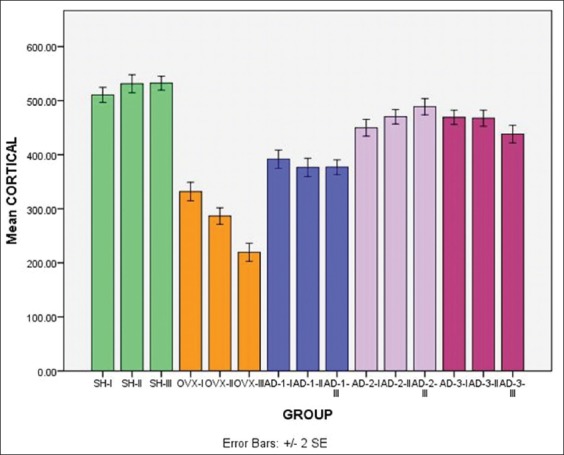
Graph illustrating the results of the cortical thickness measurements (μm) observations of 1^st^, 2^nd^, and 3^rd^ month in all treatment rat (*Rattus norvegicus*) groups.

In the group of rats was OVX that given honey therapy in month 1, the highest result was found in the AD3-I group (with a dose of 4 g/kg BW). However, it was not significantly different (p>0.05) from the AD2-I group (with a dose of 2 g/kg BW). In month 2, the highest result was found in the AD2-II group (with a dose of 2 g/kg BW). Yet, it was not significantly different (p>0.05) from the AD3-II group (with a dose of 4 g/kg BW). Different results in month 2, the highest result in month 3 was still found in the AD2-III group (with a dose of 2 g/kg BW). However, it was significantly different from the other group of rats (p<0.05). The results of the measurement of the cortical thickness femoral diaphyseal are shown in Figure-1.

Furthermore, based on the measurement of the cortical bone thickness from month 1 to month 3, there was an improvement pattern in the cortical bone thickness in the group of rats that were OVX in month 2, not significantly different from month 3. Otherwise, the OVX groups, the AD-1 group (at dose of 1 g/kg BW) and the AD-3 group (at dose of 4 g/kg BW) had a declining pattern in the cortical bone thickness from month 1 to 3. A different result was found in the AD-2 group, where there was an increasing pattern in the cortical bone thickness from month 1 to month 3 although based on results of Duncan test, there was no significant difference (p>0.05). However, the cortical bone thickness in the AD-2 (AD-2-III) group in month 3 was not significantly different (p>0.05) from that in the group of rats were not OVX in month 1 (SH-I). This suggests that honey supplements at a dose of 2 g/kg BW are effective in preventing the decreasing of the femoral diaphyseal cortical bone thickness considered as an indicator of osteoporotic bone destruction.

**Figure-2:**
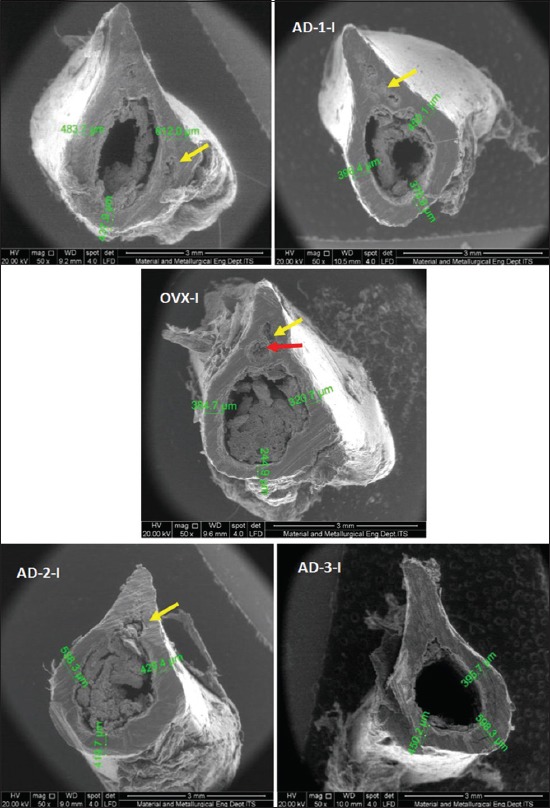
Description of microarchitecture and measurement of cortical thickness of the cross-section of femoral diaphyseal (μm) observations of the 1^st^ month in all treatment groups with scanning electron microscopy examination (50×).

In addition, determination of the damaged femoral diaphyseal area was observed descriptively based on the results of the SEM examination. Figure-2 showed a cross-section of the femoral diaphyseal bones in month 1. The figure indicates that in the group of rats was OVX without honey supplement; there were several damaged parts of the diaphyseal cortical bone area (yellow arrow). Similarly, in the group of ovariohysterectomized rats given honey supplement with the dose of 1 g/kg BW (AD-1), there were also some damaged parts although not as wide as in the OVX group. Unlike in those groups, in the group of rats was not OVX (SH) and in the group of rats was OVX with honey supplement at doses of 2 g/kg BW and 4 g/kg BW, there was no damage in the femoral diaphyseal cortical area.

**Figure-3:**
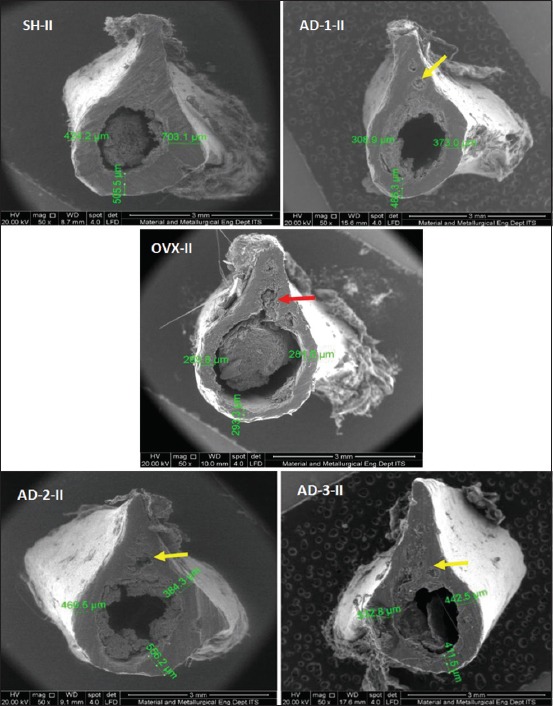
Description of microarchitecture and measurement of cortical thickness of the cross-section of femoral diaphyseal (μm) observations of the 2^nd^ month in all treatment groups with scanning electron microscopy examination (50×).

The results of the 2^nd^-month observation (Figure-3), moreover, were not much different from the results of the 1^st^-month observation. However, the damaged areas observed in the 2^nd^-month observation were more extensive than those in the 1^st^-month observation using SEM. Besides, in the AD-3 group, there was damage in some areas of the femoral diaphyseal cortical bone.

**Figure-4:**
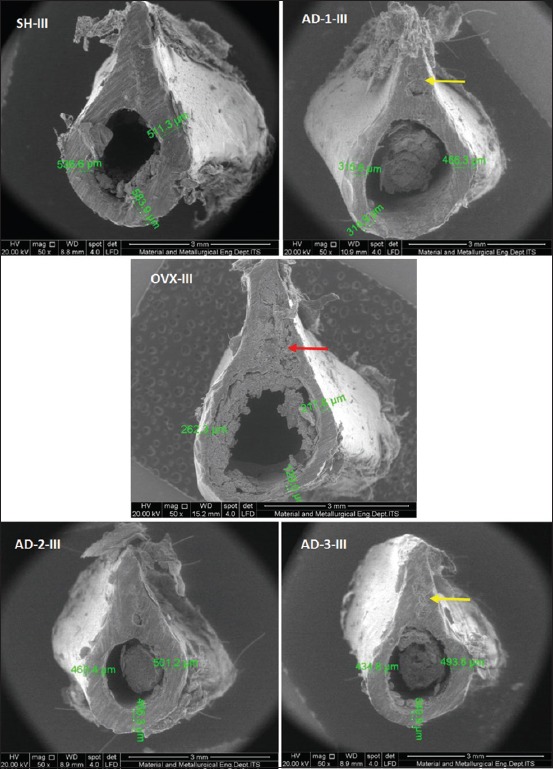
Description of microarchitecture and measurement of cortical thickness of the cross-section of femoral diaphyseal (μm) observations of 3^rd^ month in all treatment groups with scanning electron microscopy examination (50×).

The results of the 3^rd^-month observation (Figure-4), furthermore, the most extensive damage was found in the group of rats were OVX without honey supplement (red arrow). Meanwhile, in the group of rats was OVX with honey supplement began to show a decrease of the damaged areas (yellow arrow). This suggests that honey supplement is effective enough to repair bone damage occurring in months 1 and 2 after OVX. The best result was found in the AD-2 group given honey supplements at the dose of 2 g/kg BW where there was no significant damage found from month 1 to month 3.

### Bone impact strength measurement

The bone impact strength was measured with Charpy test method. The bone impact test is a test that measures bone resistance to shock load (pendulum). The principle of this impact test is to calculate both energies given by the load (pendulum) and energy absorbed by the specimen (bone). The impact index value is energy absorbed by each cross-sectional area of the test specimen [20]. The results of bone impact strength analysis are shown in Table-3.

**Table-3:** Mean and standard deviation of the impact strength measurements (Joule) observations of 1^st^, 2^nd^, and 3^rd^ month in all treatment rat (*Rattus* *norvegicus*) groups.

Group	1^st^ month	2^nd^ month	3^rd^ month
SH	76.83^f^±2.52	78.53^f^±3.13	77.69^f^±3.48
OVX	66.17^bc^±1.52	61.88^b^±1,13	53.54^a^±2.13
AD-1	68.2^cd^±2.39	69.06^cd^±3.06	67.02^cd^±4.43
AD-2	71.3^de^±1.44	75.46^ef^±2.95	77.53^f^±2.14
AD-3	79.41^f^±4.17	77.66^f^±2.57	78.08^f^±1.61

a,b,c,d,e,f different superscripts in the same column and row indicate significant differences (p<0.05)

**Figure-5:**
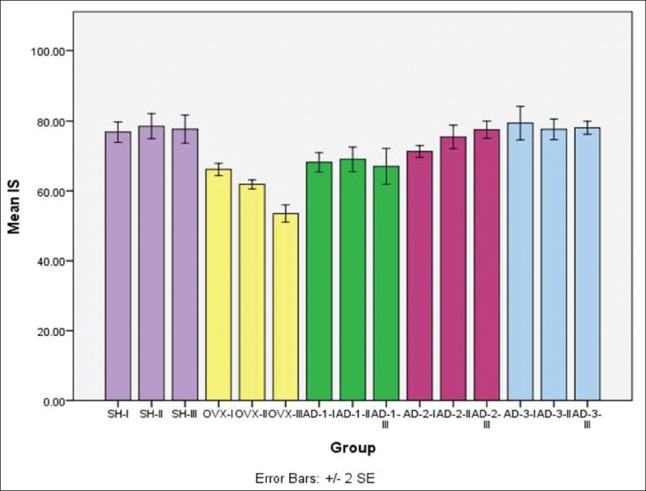
Graph illustrating the results of the impact strength measurements (Joule) observations of 1^st^, 2^nd^, and 3^rd^ month in all treatment rat (*Rattus norvegicus*) groups.

Moreover, the results of the bone impact strength measurement from month 1 to month 3 indicated that the group of rats was OVX without the administration of honey supplements had the lowest value, significantly different (p<0.05) from the other groups. However, there was no significant difference between in the OVX group in month 1 and the rats were OVX group that given honey supplement at the dose of 1 g/kg BW (AD-1) (p>0.05). The highest bone impact strength was found in the group of rats that were not OVX, significantly different from the group of rats were OVX without the honey supplement (p<0.05), but not significantly different (p>0.05) with the group of rats was OVX with administered honey supplement with dose of 2 g/kg BW (AD-2) and 4 g/kg BW (AD-3). This suggests that honey supplement at dose of 2 g/kg BW (AD-2) and 4 g/kg BW (AD-3) are able to inhibit the decreasing of bone impact strength so that the bone is stronger in resisting impact and not susceptible to fracture. Graph depicting that bone impact strength from month 1, 2, to 3 is shown in Figure-5.

## Discussion

This suggests that the effects of OVX will lead to estrogen deficiency that will affect bone health. The decrease in estrogen levels due to OVX or during menopause will also result in the loss of estrogen protection effects against oxidative stress and reactive oxygen species (ROS), as well as lead to the depletion of antioxidants in bone [21,22]. Increased ROS activity then causes excessive expressions of tumor necrosis factor -α, receptor activator of nuclear factor kappa-β ligand, and macrophage colony-stimulating factor which improve osteoclast function and lead to bone loss [23,24]. Next, oxidative stress suppresses bone formation by inhibiting osteoblast differentiation and reducing the survival of these cells. Estrogen deficiency then reduces osteoblastic activity and stimulates osteoclastic activity that eventually leads to the development of osteoporosis [21,25].

Moreover, the condition of osteoporosis usually will increase the porosity of the trabecular matrix and then will decrease in cortical thickness due to resorption on the surface of the endosteum. This condition is caused by an increase in osteoclast activity in osteoporosis patients [26]. The differences in cortical thickness can reach 20% between the bones of osteoporosis and the healthy bones [27,28].

This study shows the same results as previous research, which states that honey has an element used as anti-osteoporosis [9-11]. According to reports from previous research, honey with α-Cyclodextrin has a prebiotic effect that can decrease resorption of bone tissue [12]. The effects of honey anti-osteoporosis can occur through several mechanisms initiated by increased absorption by the intestinal lumen [29]. The mechanism is performed by lowering the pH of the intestinal lumen by gluconic acid. This decrease then increases the absorption of calcium in the gut [9].

Honey contains flavonoid compounds, more than 150 polyphenol compounds, including phenolic acids, flavonoids, flavonols, catechins, and cinnamic acid derivatives [13]. Flavonoids contained in honey have a role in protecting osteoporosis through five mechanisms, namely reducing bone loss through antioxidant and anti-inflammatory actions, increasing osteoblastogenesis, suppressing osteoclastogenesis, as well as working on osteoimmunological action [14].

In addition, based on results of the phytochemical scanning test on the active ingredients contained in the honey forest (*A. dorsata*), there are alkaloid content, triterpenoid/free steroid, unsaturated steroids, flavonoids, polyphenols, and tannins. This proves that forest honey (*A. dorsata*) has activity as antiosteoporosis due to the active ingredient contained in it.

Flavonoids, moreover, can increase the proliferation and differentiation of osteoblasts [30]. Flavonols contained in honey can also induce apoptosis and decrease ROS activity in mature osteoclasts. Flavonols then will directly bind to the estrogen receptor (ER)-α and ER-β [9]. Besides, flavonoids can decrease the activity of secretory tartrate-resistant acid phosphatase (sTRAP) in osteoclasts. TRAP 5b is a marker for bone resorption activity. Flavonoids can also increase the proliferation of osteoblast cells tested using Microtetrazolium (MTT) assay [31]. Other studies also showed proline-rich membrane anchor-linked acetylcholinesterase (AChE), a special form of AChE in MG63 osteosarcoma and primary osteoblast cells, increased in flavonoid-induced osteoblast differentiation. AChE is secreted by osteoblasts that exhibit cell proliferation [32].

Furthermore, based on the results of the Pearson Correlation test, the results of this bone impact measurement were correlated very strongly with the results of the SEM examination aimed to observe the microarchitecture and thickness of the femoral diaphyseal cortical bone with a correlation coefficient value of 0.894 and a significant correlation value of 0.000. Similarly, Mubeen *et al*. [33] stated that the major structural determinants of bone mechanical strength include the thickness and porosity of the cortical bone, and then the shape, width, connectivity as well as anisotropy of the trabecular bone. Ruiz *et al*. [34] argued that bone impact strength is affected by quality (bone structure and matrix as well as bone microarchitecture) and mineral density of bone. Bone quality is determined by three important factors, namely cortical bone size, bone turnover, and bone geometry (microarchitecture). Meanwhile, the quantity of bone is determined by the bone density that is the number of minerals in grams per volume.

Based on the above statement it can be understood that cortical bone thickness and microarchitecture greatly affect the bone mechanical properties, in addition to other factors, the speed of bone burn turnover and bone density that also need to be taken into account. Yang *et al*. [35] also stated that bone strength is not only determined by the mineral content of bone mass but also determined by structural characteristics of bone, such as size, shape, and arrangement of bone architecture.

The decrease in bone mass is, in fact, identifiable from bone density. Besides, it can also be predicted from bone structural changes, such as changes in cortical and trabecular mass. Changes in the mass of cortical and trabecular regions affect bone strength due to differences in mineral content that determines the function of both regions. According to Clarke [36], the cortical part serves mechanically, while the trabecular part serves metabolically. Trabecula has greater metabolic activity, more mineral changes than cortical ones so that it can trigger predisposition for bone mass deficiency. In other words, cortical and trabecular bone thicknesses as structural parts of the bone can reflect bone strength since bone mass changes can also be identified from changes in cortical and trabecular bone sizes, and decreased bone mass can lead to decreased bone mechanical strength.

Similarly, Chen *et al*. [37] suggest that in osteoporosis conditions there will be an increase in trabecular matrix porosity and a decrease in cortical bone thickness which will lead to a decrease in bone mass subsequently resulting in a decrease in bone mechanical strength.

## Conclusion

Finally, it can be concluded that the provision of honey supplements in the group of ovariohysterectomized rats can inhibit the decreasing of the cortical bone thickness and repair damage in microarchitecture to generate bone impact strength. As a result, bones are not easily broken.

## Authors’ Contributions

We declare that this work was done by the authors named in this article and all liabilities pertaining to claims relating to the content of this article will be borne by them. ISY supervised the whole study. ISY and HP designed the study. LN and HP performed the OVX procedure, ISY and GAY analyzed the data and wrote the manuscript. The final manuscript has been read and approved by all the authors.
